# Rich essential properties of Si-doped graphene

**DOI:** 10.1038/s41598-020-68765-x

**Published:** 2020-07-21

**Authors:** Duy Khanh Nguyen, Ngoc Thanh Thuy Tran, Yu-Huang Chiu, Godfrey Gumbs, Ming-Fa Lin

**Affiliations:** 1 Laboratory of Applied Physics, Advanced Institute of Materials Science, Ton Duc Thang University, Ho Chi Minh City, Vietnam; 2 Faculty of Applied Sciences, Ton Duc Thang University, Ho Chi Minh City, Vietnam; 3 Hierarchical Green-Energy Materials (Hi-GEM) Research Center, National Cheng Kung University, Tainan, Taiwan; 4 Department of Applied Physics, National Pingtung University, Pingtung, Taiwan; 5 Department of Physics and Astronomy, Hunter College of the City University of New York, New York, USA; 6 Department of Physics/QTC/Hi-GEM, National Cheng Kung University, Tainan, Taiwan

**Keywords:** Materials science, Theory and computation

## Abstract

The diverse structural and electronic properties of the Si-adsorbed and -substituted monolayer graphene systems are studied by a complete theoretical framework under the first-principles calculations, including the adatom-diversified geometric structures, the Si- and C-dominated energy bands, the spatial charge densities, variations in the spatial charge densities and the atom- and orbital-projected density of states (DOSs). These critical physical quantities are unified together to display a distinct physical and chemical picture in the studying systems. Under the Si-adsorption and Si-substitution effects, the planar geometric structures are still remained mainly owing to the very strong C–C and Si–C bonds on the honeycomb lattices, respectively. The Si-adsorption cases can create free carriers, while the finite- or zero-gap semiconducting behaviors are revealed in various Si-substitution configurations. The developed theoretical framework can be fully generalized to other emergent layered materials. The Si-doped graphene systems might be a highly promising anode material in the lithium-ion battery owing to its rich potential properties.

## Introduction

Carbon atoms can form three-dimensional (3D) diamond^1^, 3D graphites^2^, two-dimensional (2D) graphene with the pure sp$^2$ carbon atoms^3^, 2D graphdiyne with the unique sp–sp$^2$ carbon atoms^4^, one-dimensional (1D) graphene nanoribbons^5–7^, 1D carbon nanotubes^8–10^, zero-dimensional (0D) carbon toroids^11,12^, 0D C$_{60}$-related fullerenes^13^, and 0D carbon onions^14^. The versatile morphologies directly indicate the peculiar chemical bondings, in which all carbon-created systems possess ${sp^2}$-bonding surfaces except for the ${sp^3}$ bondings in diamond. Specifically, the few- and multi-layer graphene systems have been manufactured using the various methods^15,16^ since the first experimental observation in 2004 by mechanical exfoliation. Up to now, they clearly exhibit plenty of remarkable fundamental properties due to the hexagonal symmetry, the nanoscaled thickness, and the distinct stacking configurations, such as semiconducting and semi-metallic behaviors^17,18^, anomalous quantum Hall effects^19^, diverse magnetic quantizations^20–23^, rich Coulomb excitations and decays^24–28^, different magneto-optical selection rules^29–31^, the exceedingly high mobility of charge carriers^32,33^, and the largest Young’s modulus of materials ever tested^34^. To induce the novel phenomena and extend the potential applications, the electronic properties can be easily modulated by the layer number^35,36^, stacking configuration^37–39^, mechanical strain^40,41^, sliding^42^, electric and magnetic field^43,44^, atom adsorption^45–48^ and substitution^49–51^. This paper mainly focuses on the latter two factors.

**Table 1:** Energy gap $$ E_g$$ (eV)/metal (M)/semimetal (SM); C–C bond length (Å), Si–C bond length (Å), and Si height (Å) for Si-absorbed and Si-substituted graphene.

Configuration	Ratio of Si and C	Percentage	$E_g^{d(i)}$ (eV) semimetal(SM) metal(M)	C-C(Å)	Si-C (Å)	Si height (Å)
Pristine	X	X	$E_g^{d}=0$	1.420	X	X
Adsorption	Si:C = 6:6	$100\%$	SM	1.492	2.535	2.422
	Si:C = 3:6	$50\%$	SM	1.490	2.514	2.400
	Si:C = 1:6	$16.6\%$	M	1.456	2.138	2.000
	Si:C = 1:8	$12.5\%$	M	1.450	2.118	1.989
	Si:C = 1:24	$4\%$	M	1.454	2.125	1.996
Substitution	Si:C = 3:3	$100\%$	$E_g^{i}=2.56$	X	1.780	X
	Si:C = 2:4	$50\%$ ortho	$E_g^{d}=0.04$	1.478	1.833	X
	Si:C = 2:4	$50\%$ meta	$E_g^{d}=0.56$	1.465	1.829	X
	Si:C = 1:5	$$20\%$$	$$ E_g^{d}=0$$	1.351	1.621	X
	Si:C = 1:17	$$5.8\%$$	$$ E_g^{d}=0$$	1.372	1.631	X
	Si:C = 1:23	$$4.3\%$$	$$ E_g^{d}=0$$	1.379	1.649	X

**Figure 1:**
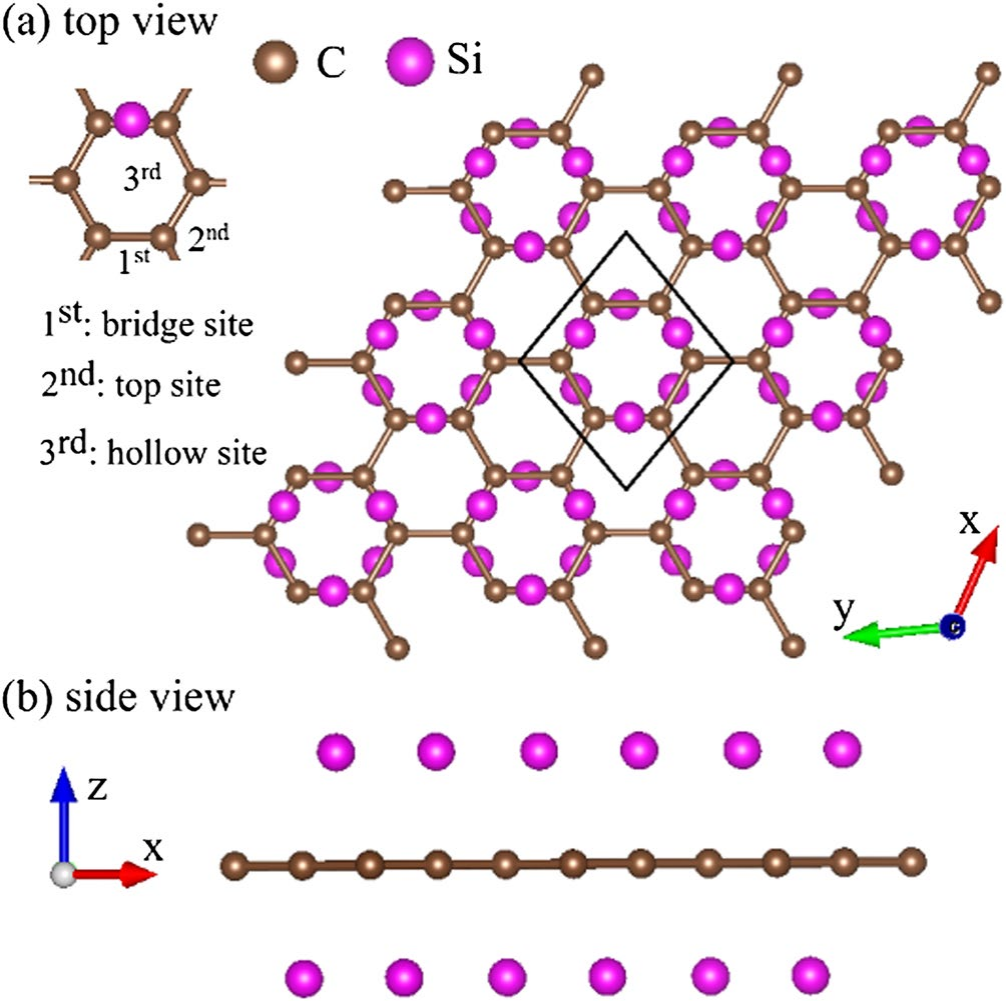
Geometric structure of the 100$\%$ Si-adsorbed graphene for (**a**) top view and (**b**) side view. The bridge site is the most optimal adsorption position among the top and hollow sites^94^.

**Figure 2:**
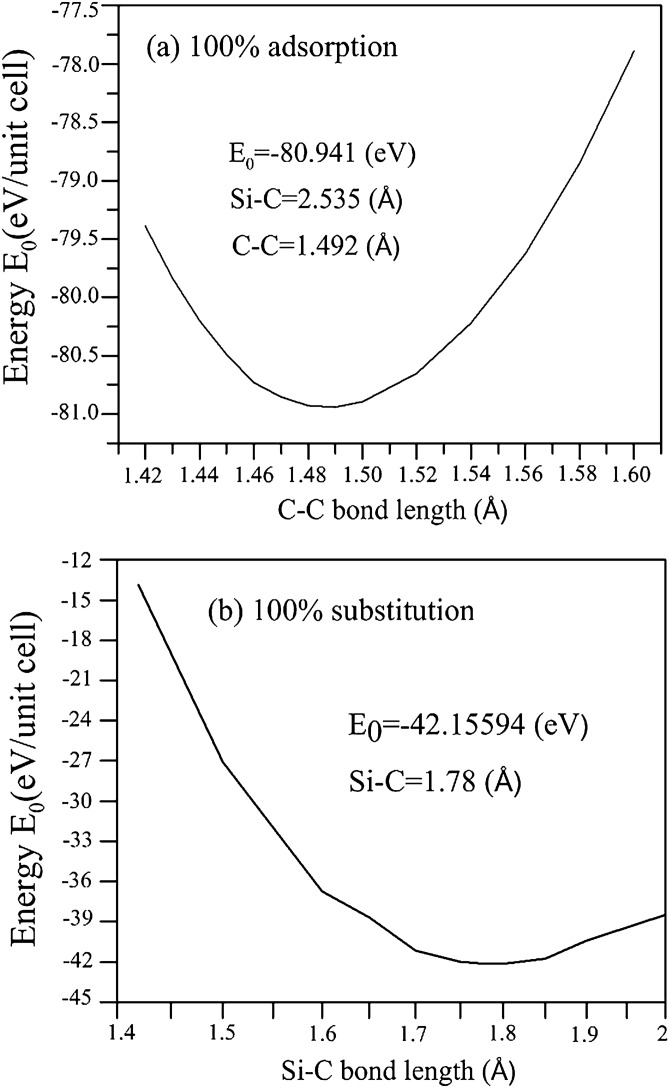
Dependence of total ground state energy on C–C and Si–C bond lengths for (**a**) 100$\%$ Si double-side adsorption and (**b**) 100$\%$ Si substitution, respectively^95^. [OriginPro 2015 SR2, version number: 272, https://www.originlab.com/index.aspx?go=Support&pid=3168].

**Figure 3:**
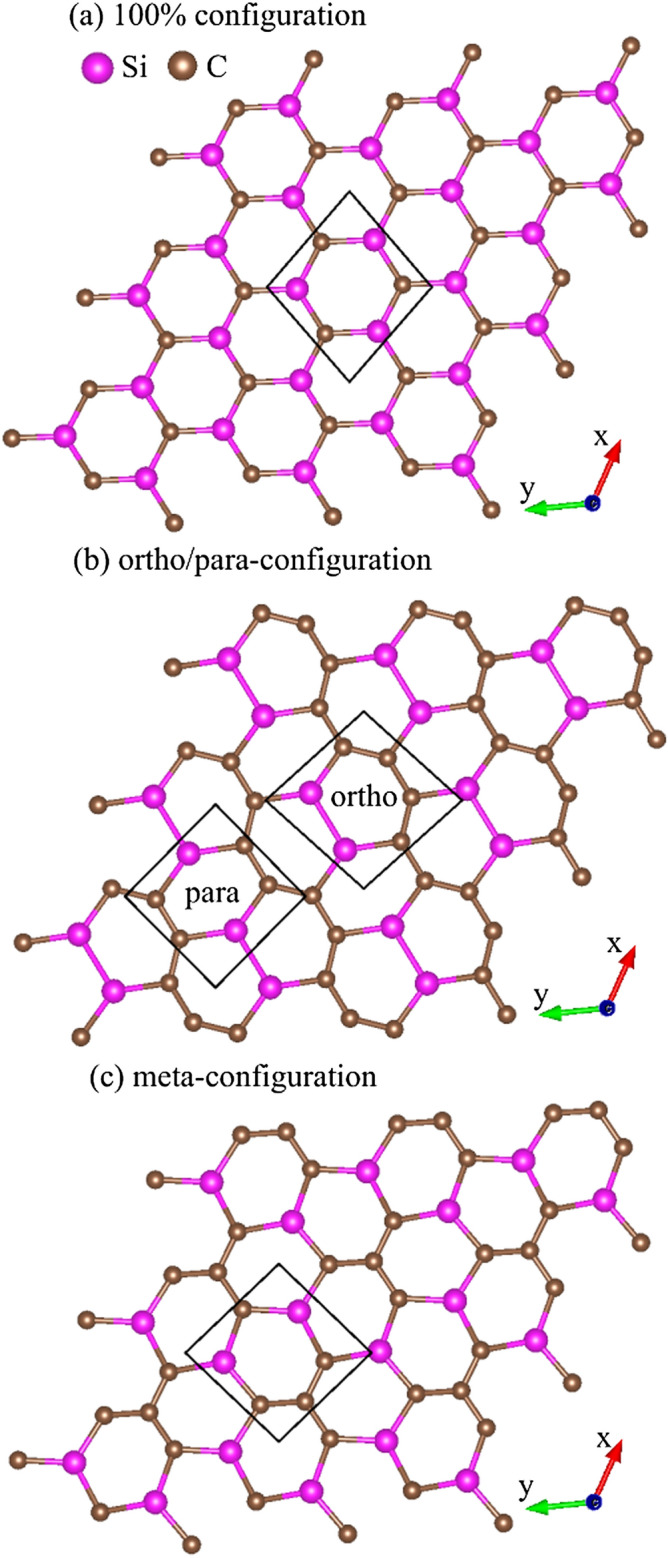
Geometric structures of Si-substituted graphene systems for (**a**) 100$\%$ Si substitution, (**b**) 50$\%$ Si ortho-/para-substitution and (**c**) 50$\%$ Si meta-substitution^94^.

**Figure 4:**
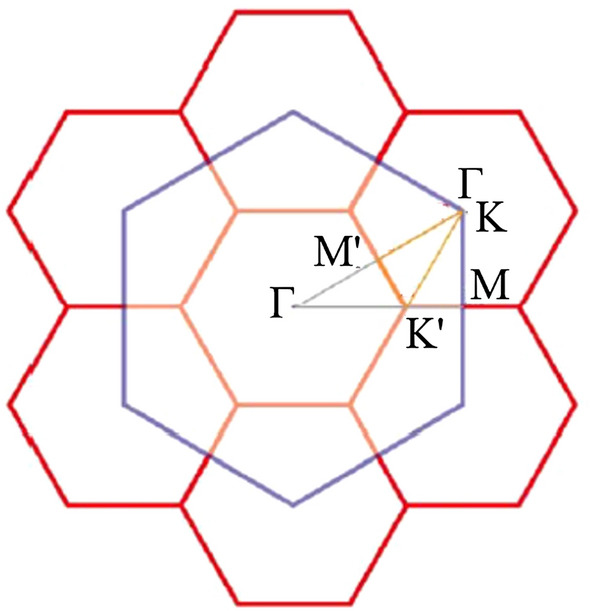
Diagram of the first Brillouin zones for various unit cells^94^.

**Figure 5:**
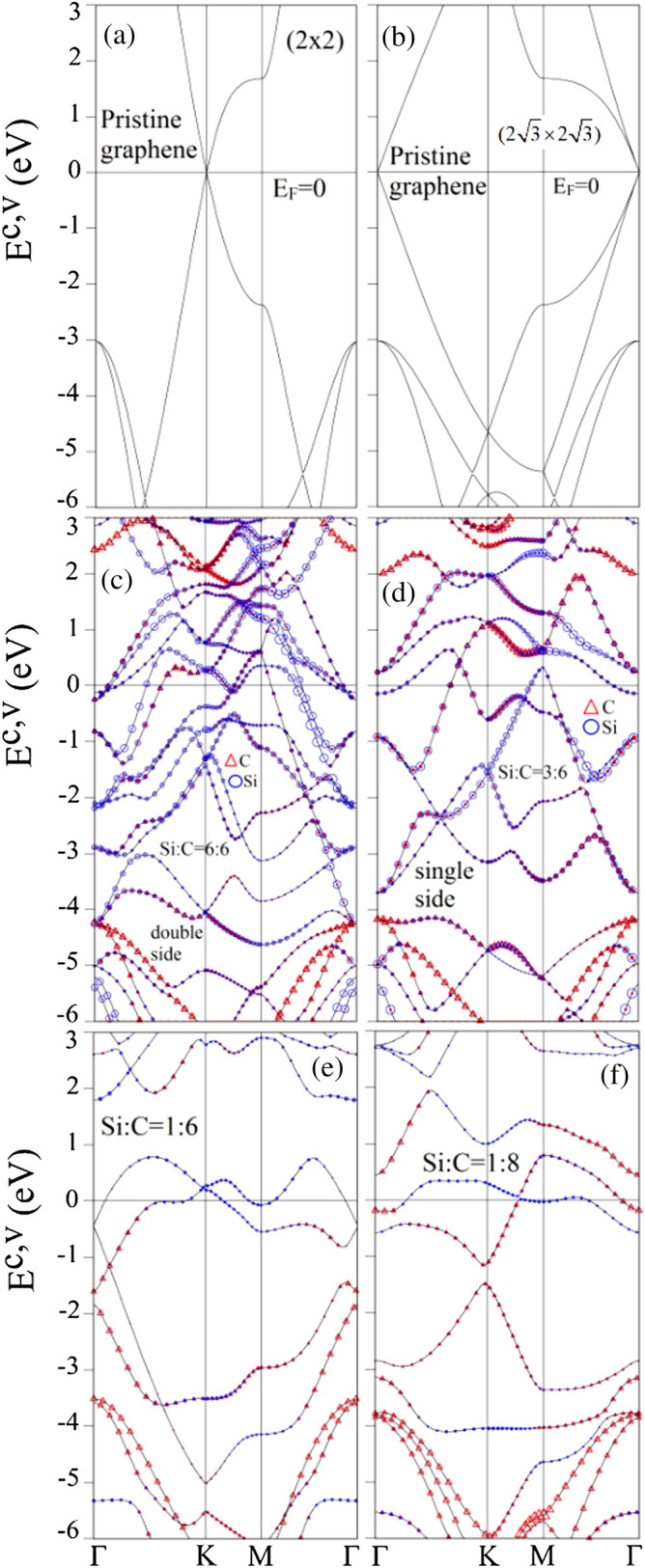
Electronic band structures with the dominance of C-host and Si-guest atoms for Si-adsorbed graphene systems: (**a**) pristine case, (**b**) pristine graphene under the enlarged unit cell identical to the 100$\%$ case, (**c**) $100\%$ Si double-side adsorption, and (**d**) $100\%$ Si single-side adsorption, (**e**) $16.6\%$ Si adsorption, and (**f**) $12.5\%$ Si adsorption. Red triangle and blue circle illustrate for the dominance of C-host and Si-guest atom, respectively^95^. [OriginPro 2015 SR2, version number: 272, https://www.originlab.com/index.aspx?go=Support&pid=3168].

**Figure 6:**
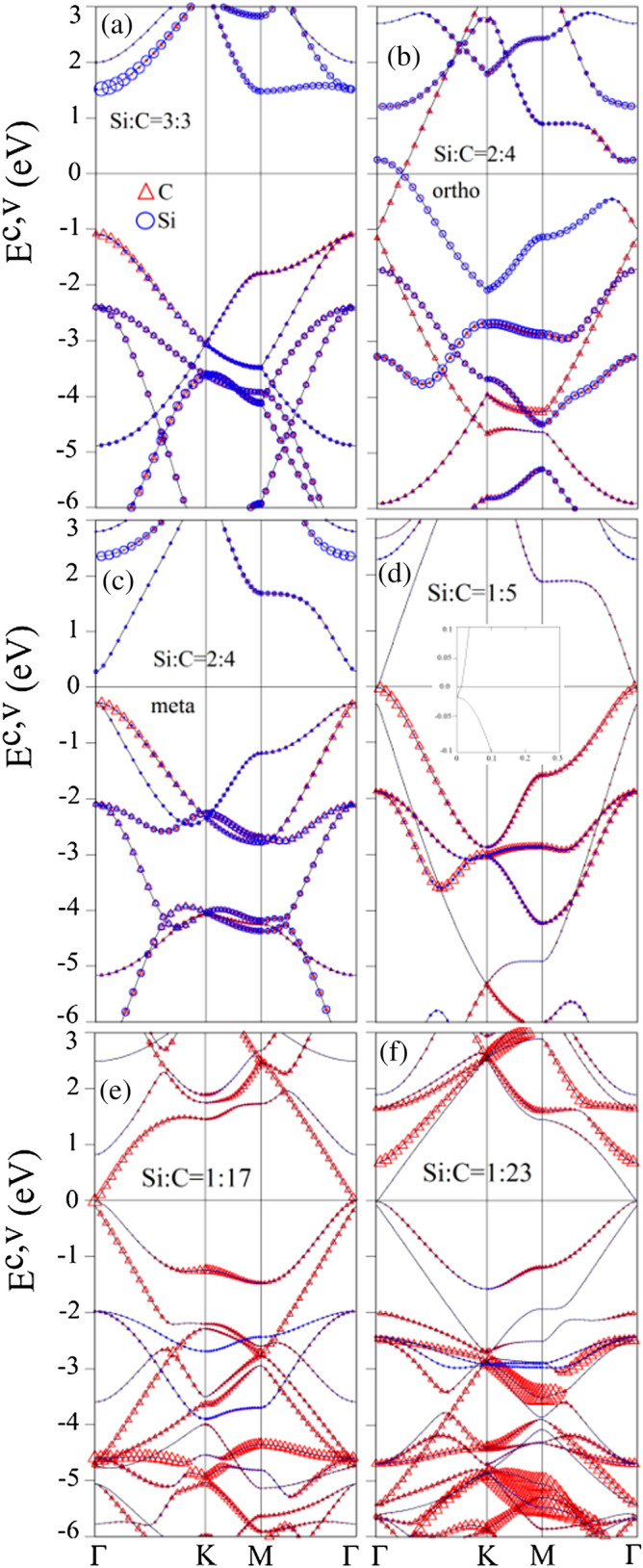
C- and Si-dominated valence and conduction bands of Si-substituted graphene systems: (**a**) 100$\%$ Si substitution, (**b**) 50$\%$ Si ortho-substitution, (**c**) 50$\%$ Si meta-substitution, (**d**) 20$\%$ Si substitution, (**e**) 5.8$\%$ Si substitution, (**f**) 4.3$\%$ Si substitution. Red triangle and blue circle represent for the dominance of the C-host and Si-guest atom, respectively^95^. [OriginPro 2015 SR2, version number: 272, https://www.originlab.com/index.aspx?go=Support&pid=3168].

**Figure 7:**
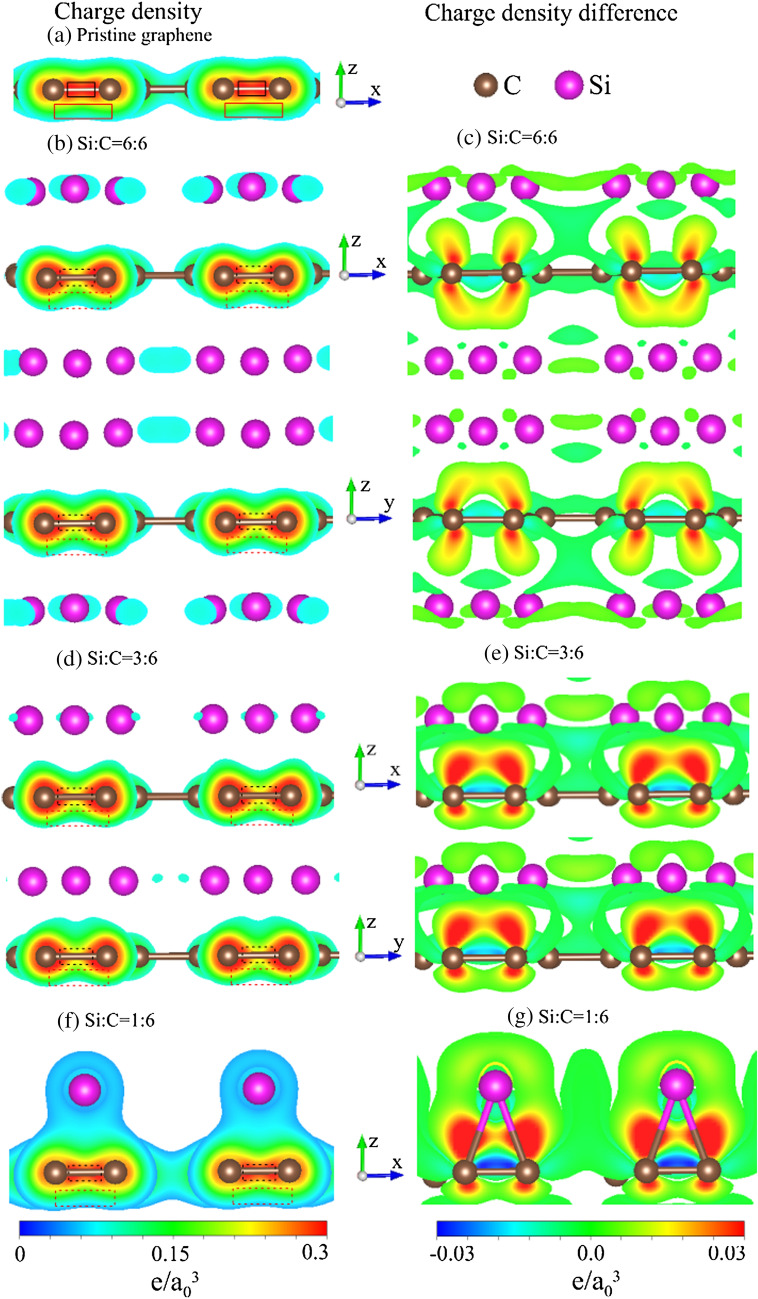
Spatial charge density for (**a**) pristine graphene; spatial charge density and charge density difference situated at the left-hand side and the right-hand side, respectively, for (**b**) and (**c**) 100$\%$ Si double-side adsorption, (**d**) and (**e**) 100$\%$ Si single-side adsorption and (**f**) and (**g**) $16.6\%$ Si adsorption^94^.

**Figure 8:**
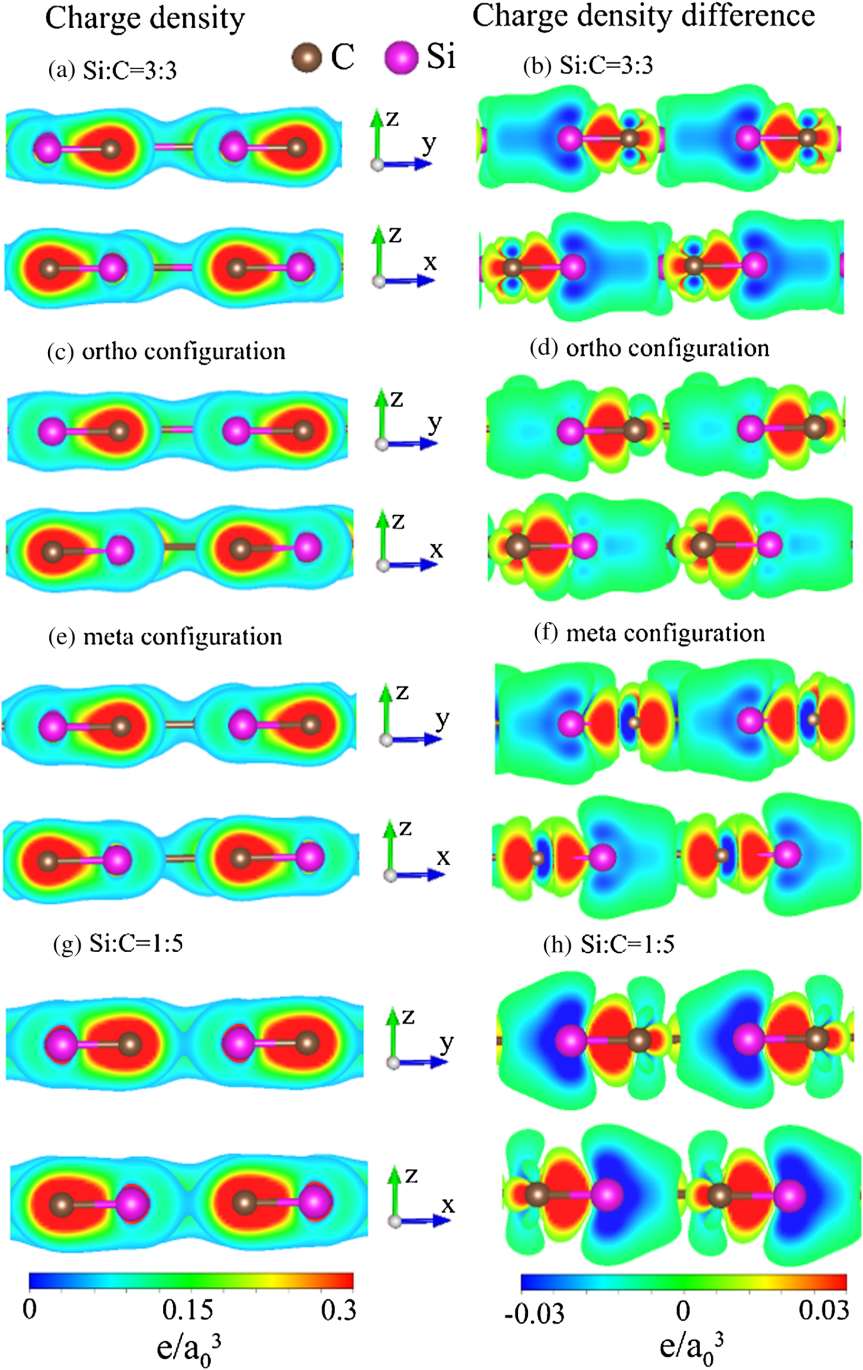
Similar plot as Fig. 7, but shown for Si-substituted graphene systems for (**a**) and (**b**) 100$\%$ Si substitution, (**c**) and (**d**) 50$\%$ Si ortho-substitution, (**e**) and (**f**) 50$\%$ Si meta-substitution and (**g**) and (**h**) 20$\%$ Si substitution^94^.

**Figure 9:**
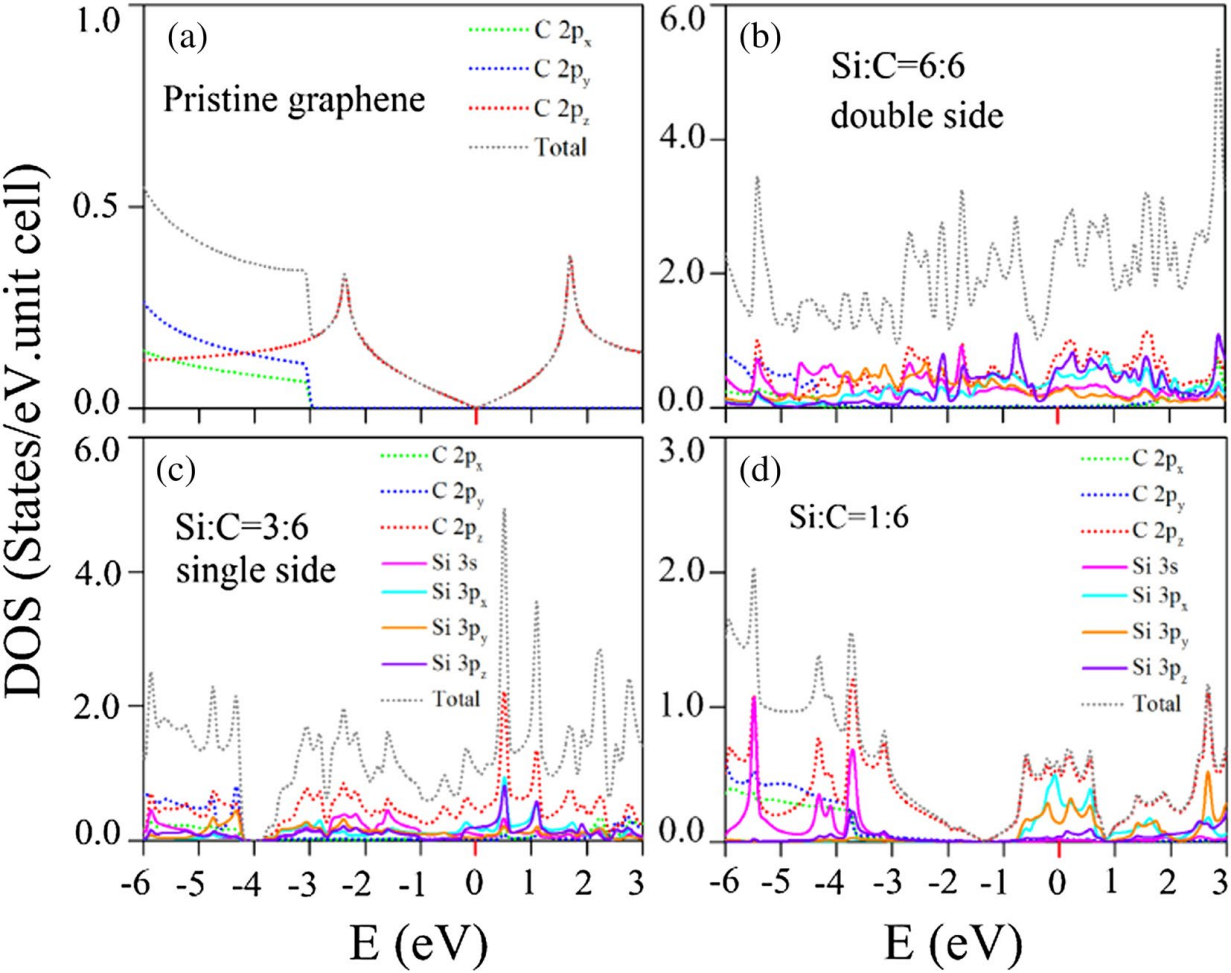
Orbital-projected density of states for Si-adsorbed graphene systems: (**a**) pristine case, (**b**) 100$\%$ Si double-side adsorption, (**c**) 100$\%$ Si single-side adsorption, and (**d**) 16.6$\%$ Si adsorption^95^. [OriginPro 2015 SR2, version number: 272, https://www.originlab.com/index.aspx?go=Support&pid=3168].

**Figure 10:**
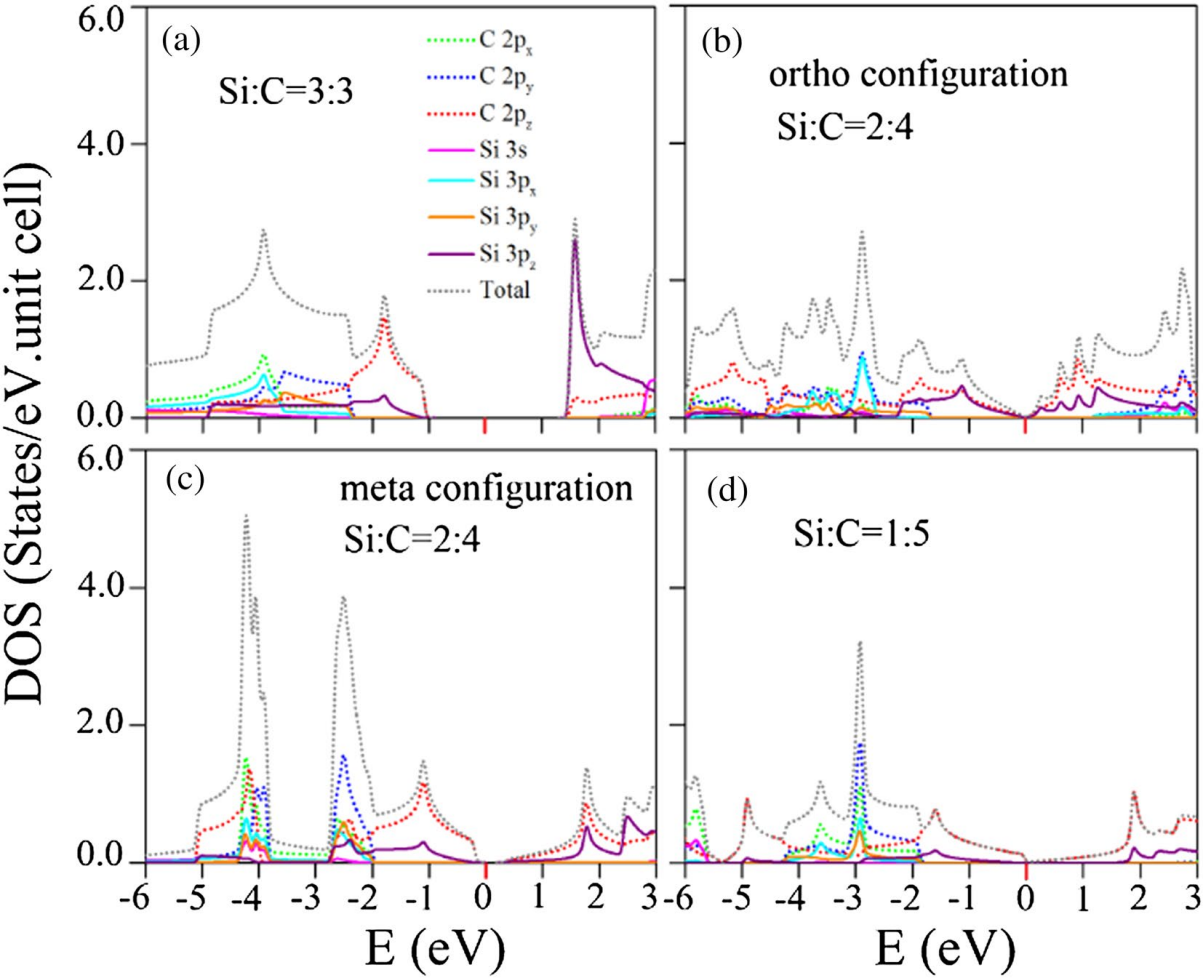
Similar plot as Fig. 9, but displayed for Si-substituted graphene systems: (**a**) 100$\%$ Si substitution, (**b**) 50$\%$ Si ortho-substitution, (**c**) 50$\%$ Si meta-substitution, and (**d**) 20$\%$ Si substitution^95^. [OriginPro 2015 SR2, version number: 272, https://www.originlab.com/index.aspx?go=Support&pid=3168].

How to modulate the fundamental properties becomes one of the main-stream topics in materials science, chemistry, physics and engineering. The chemical modification is the most effective method. Pristine graphene has a rather strong $\sigma$ bonding of 2s, 2p$_x$, and 2p$_y$ orbitals in a honeycomb lattice. This system creates a quite active chemical environment since each carbon contributes one perpendicular 2p$_z$ orbital as a dangling bond. That is to say, the host carbon atoms can bond with the various guest atoms (adatoms)^52,53^, molecules^54^ and even functional groups^55^. To date, many theoretical^56^ and experimental research papers on surface adsorptions have been published^57^. The previous results show adsorption-diversified physical and chemical phenomena, such as an opening of the energy gap^58^, semiconductor-metal transitions^59–62^, the absence/recovery of a Dirac-cone structure^63^, spin-splitting energy bands due to specific adatoms^64^, single- or multi-orbital hybridizations in C-adatom bonds shown in the spatial charge density and atom- and orbital-decomposed DOSs^65^, and the shift of the Dirac cone of graphene field effect transistors (GFETs) under doping with the polyethylenimine (PEI) molecule^66^. It is well known that graphitic systems are the most efficient anode material in Li$^+$-based batteries. Possibly, the silicon doping can play an important role to enhance the battery efficiency^67^ and put to use for nano-scaled applications. Another chemical modification is the direct doping, the substitution of carbon host atoms by guest adatoms in the honeycomb lattice. For example, diversifying the physical and chemical properties of graphene by the silicon doping effects^68^, modifying the properties of graphene by exactly positioning atomic dopants of nitrogen or phosphorus^69,70^. Besides, the B$_x$C$_y$N$_z$ compounds have been successfully synthesized in the stable structures of 1D nanotubes^71^ and 2D layers^72^. Apparently, the essential properties are predicted to present drastic changes under the adatom dopings, e.g., in the electronic structures^73^, optical absorption spectra^74^ and transport properties^75^. The feature-rich properties of Si-adsorbed and Si-substituted graphene systems are worthy of a complete systematic study, especially for the critical differences in the orbital hybridizations between the weak and strong Si–C bonds.

In this paper, a complete theoretical framework, which is developed by the first-principles calculations, is utilized to fully explore the feature-rich properties of the Si-adsorbed and Si-substituted graphene systems. The concise chemical and physical pictures and the multi-orbital hybridizations will be proposed to explain the adatom diversified geometric structures, electronic energy spectra, spatial charge densities, and atom- and orbital-decomposed density of states. That is, all the calculated results are consistent with one another under such mechanisms. Furthermore, they are responsible for the semiconductor-metal transitions after the guest-atom adsorptions and the creation of energy gaps in the substitution cases. The theoretical predictions can be thoroughly verified by the various experimental measurements, including the scanning tunneling microscopy (STM)/transmission electron microscopy (TEM), angle-resolved photoemission spectroscopy (ARPES) and scanning tunneling spectroscopy (STS).

## Result and discussion

### Geometric structure

Monolayer graphene has a planar geometry with a honeycomb lattice, being different from the buckled structures in layered silicene^76^, germanene^77^, and tinene^78^. Apparently, this crystal is formed by the very strong $\sigma$ bonding of 2s, 2p$_x$, and 2p$_y$ orbitals, and the weak $\pi$ bond of 2p$_z$ orbitals perpendicular to the graphitic plane. However, the other group-IV systems, with the buckled structures, are stabilized by the optimal competition between the ${sp^2}$ and ${sp^3}$ chemical bondings. The bond length among all the group-IV systems remains shortest for the C–C (1.42 Å in Table 1). After the Si adsorption on the graphene surface, the bridge site is the most optimal adsorption position among the top and hollow sites, as shown in Fig. 1. The hexagonal honeycomb lattice of carbon atoms remains a planar structure, while C–C bond lengths are lengthened ${\sim \,1.45}$–1.49 Å under various adatom concentrations, as shown in Table 1 and Fig. 2a. Part of the carbon electrons participate in the multi-orbital hybridizations of Si–C bonds, leading to the weakened C–C bondings. As for the Si-substituted cases, the silicon-carbon honeycomb lattices remain planar structures, indicating sufficiently strong quasi-$\sigma$ bonds due to the ${sp^2}$–${sp^2}$ multi-orbital hybridizations in the Si–C bonds. The Si–C and C–C bond lengths are, respectively, ${\sim \,1.62}$–1.83 Å and ${\sim \,1.35}$–1.47 Å under various substitution cases (Table 1). The 1:1 substituted system (Fig. 3a), the pure silicon–carbon compound, has an optimal Si–C bond length of 1.78 Å as shown in Fig. 2b, which is much longer than that C–C of 1.42 Å in pristine graphene. This will lead to a great decrease of the strength in quasi-$\sigma$ bonding, being further identified by the largely reduced charge density in the Si–C bonds. In addition, the guest-atom distribution configurations could be classified into three kinds under specific concentrations lower than 50$\%$ shown in Fig. 3b,c, namely, the ortho-, para- and meta-substitution cases. However, the former two are degenerate just at 50$\%$ (Fig.  3b).

### Electronic band structure

A diagram of the first Brillouin zones for various unit cells is presented in Fig. 4, in which it is very useful to understand the change in the band structures under elongation effects and the highly symmetric $\Gamma$-K-M-$\Gamma$ points in the band structures are represented by using the k-points sampling in such first Brillouin zones. The pristine monolayer graphene systems exhibit unusual band structures, as clearly indicated in Fig. 5a,b. The occupied valence bands are asymmetric about the unoccupied ones, mainly owing to the multi-orbital $\sigma$ bondings. The low-lying valence and conduction bands, which are initiated from the K and K$^\prime$ valleys (the corners of the first Brillouin zone), are linearly intersecting there. There exist the isotropic Dirac-cone structures at low energy, in which the Fermi level (${E_F=0}$) just crosses at the Dirac point. Apparently, this system belongs to a zero-gap semiconductor because of the vanishing density of state at ${E_F}$. The low-energy bands mainly come from the $\pi$ bondings of the perpendicular C-${2p_z}$ orbitals. The Dirac-cone structure, being due to the hexagonal symmetry, is predicted to display a lot of unusual phenomena, e.g., diverse magnetic quantizations^79^, Hall effects^80^ and optical properties^81^, being consistent with the experimental measurements^82^. The linear energy dispersions will gradually become parabolic ones as the state energy of ${|E^{c,v}|}$ grows, or the wave vector deviates from the K/K$^\prime$ point. Specifically, the middle-energy parabolic valence and conduction bands present the saddle points at M point, respectively, corresponding to ${\sim -2.4}$ eV and 1.8 eV. Such critical points in the energy-wave-vector space could be regarded as the significant band-edge states in creating the important van Hove singularities. That is to say, they are thus expected to induce special structures in the essential physical properties. The $\sigma$ valence bands at the deeper energy come into existence at ${E^v\sim \,-3}$ eV from the $\Gamma$ point, being regarded as the extreme point of parabolic energy dispersion. Their electronic states are formed by the 2s, 2p$_x$, and 2p$_y$ orbitals of carbon atoms, or the very strong $\sigma$ bondings on the graphene plane.

The electronic band structure of the pristine systems is fully changed under the Si adsorptions. The band asymmetry about the Fermi level becomes more obvious. For the $100\%$ Si double-side adsorption, as shown in Fig. 5c, there exist some valence and conduction bands simultaneously intersecting with the Fermi level (${E_F=0}$) so that this system exhibits semi-metallic behavior. Apparently, the distorted Dirac-cone structure appears near the $\Gamma$ point. The separation of valence and conduction Dirac points could reach ${\sim \,0.5}$ eV. The electronic energy spectrum is highly anisotropic energy one along $\Gamma$M and $\Gamma$K. The occupied electronic states come to exit between the Fermi level and the bottom of the conduction-band states. This clearly indicates the creation of free conduction electrons by the effective adatom dopings. On the other hand, free holes are generated in the unoccupied valence states along M$\Gamma$ and K$\Gamma$ lines. As a result, it is difficult to identify Si-adsorbed graphene as a n-type or p-type system. However, this system belongs to a 2D semimetal since it has a finite density of states at the Fermi level arising from the crossing valence and conduction subbands. The above-mentioned unusual band structures are closely related to the very strong competition of orbital hybridizations in Si–C and C–C bonds. Also, some drastic changes in electronic structures are revealed in the $100\%$ Si single-side adsorption case, as clearly indicated by a comparison of Fig. 5d,c. The adsorption of silicon adatoms induces the free electrons and holes simultaneously, similar to the double-side case (Fig. 5c). The low-lying conduction bands near the $\Gamma$ point and the vacant valence bands along M$\Gamma$ and K$\Gamma$ lines reduced in number. That is to say, the number of energy bands intersecting with the Fermi level declines for a further decrease of the Si concentration, and the 2D free carrier density behaves so. As to both adsorption cases, carbon host atoms and silicon guest ones make significant contributions to the electronic structures of the whole energy range, in which their dominances are obviously displayed by the red triangles and blue circles. These important results mean that there exist non-negligible multi-orbital hybridizations in Si–C, C–C and Si–Si bonds. Furthermore, there is an obvious difference from the aforementioned $100\%$ adsorption cases when the concentration decreases (Fig. 5e,f). That is, only the valence energy bands intersect with the Fermi level. This indicates that unoccupied electronic states between the Fermi level and the top of the valence-band states all belong to free holes. As a result, these systems can be regarded as the p-type metal. Especially, the anisotropic Dirac cone structure without/with separation (Fig.  5e,f) between the $\Gamma$ and K points appears below the Fermi level, respectively.

The atom- and orbital-dominated energy bands are worthy of a closer examination. For 100$\%$ Si double-side and single-side adsorption cases, most of energy bands are co-contributed by C-host and Si-guest atoms with part of them mainly coming from either the former or the latter. In general, the low-lying and middle-energy valence and conduction bands are dominated by the Si adatoms. The percentage of atom contribution is about 4:1 (2:1) in the Si-100$\%$ (Si-50$\%$) adsorption case, as shown in Fig. 5c (Fig. 5d), being estimated from the $sp^3 -p$ bonding in the Si–C bond. The four orbitals (3s, 3p$_x$, 3p$_y$ and 3p$_z$) of silicon and the single 2p$_z$ orbital of carbon make important contributions to such energy bands. Apparently, there are few $\sigma$ valence bands of (2p$_x$, 2p$_y$) orbitals near the $\Gamma$ point; they belong to the concave-downward energy dispersions at ${E^v\sim \,-4.1}$ eV and $-4.2$ eV for the 100$\%$ adsorption cases (Fig. 5c,d). It should be noted that the $\sigma$ valence bands come into existence at ${E^v\sim \,-3.0}$ eV (Fig. 5a,b). As the concentration declines, the Si and C co-dominated energy bands only appear at specific energies, while other bands are mainly dominated by C atoms, as indicated in Fig.  5e,f for $16.6\%$ and $12.5\%$ adsorption cases, respectively. Also, the $\sigma$ valence bands of (2p$_x$, 2p$_y$) orbitals situate at ${E^v\sim \,-3.5}$ eV and ${E^v\sim \,-3.2}$ eV (Fig. 5e,f). This clearly illustrates that the shorter C–C bond lengths under lower concentrations, as compared with the 100$\%$ cases (Fig. 5c,d).

Both silicon and alkali adatoms can create free carriers, while their band properties are quite different from each other. The alkali-adsorbed graphene systems present approximately rigid energy bands and a few Li-dominated conduction bands, clearly indicating the blue shift of the Fermi level. Their free carriers purely originate from the electron charge transfer from the outermost s orbital of each alkali adatom to the carbon host atom. Furthermore, the atom- and orbital-projected densities of states clearly show the weak, but significant s-p$_z$ orbital hybridization in every alkali-carbon bond. On the other hand, the free electrons and holes in the Si-adsorbed cases are associated with a strong overlap of valence and conduction bands so that the shift of $E_F$ is very difficult to characterize in value except for the low concentrations, in which the Fermi level exhibits a red shift. There are a lot of extra Si-dominated and (Si, C)-co-dominated energy bands in the whole energy spectrum, especially for those crossing at the Fermi level. The complicated chemical bondings are deduced to survive in Si-adsorbed graphene systems, in which they cover the 2p$_z$-(3s, 3p$_x$, 3p$_y$, 3p$_z$), (3s, 3p$_x$, 3p$_y$, 3p$_z$)-(3s, 3p$_x$, 3p$_y$, 3p$_z$) and (2s, 2p$_x$, 2p$_y$)-(2s, 2p$_x$, 2p$_y$) multi-orbital hybridizations in the C–Si, Si–Si and C–C bonds. Most importantly, the above-mentioned differences obviously illustrate the adatom-adsorption-induced diverse phenomena and the critical mechanisms in determining the fundamental properties.

Electronic band structures obviously change in the presence of Si substitutions, as clearly indicated in Fig. 6a–f. The asymmetry of valence and conduction bands about $E_F=0$ is greatly enhanced after various substitutions, e.g., 100$\%$ case in Fig. 6a, 50$\%$ case in Fig. 6b,c, 20$\%$ case in Fig. 6d, 5.8$\%$ case in Fig. 6e, and 4.3$\%$ case in Fig. 6f. The substitution and adsorption cases sharply contrast with each other in Figs. 5 and 6. First, all the substitution configurations and concentrations show the semiconducting behavior with a finite or vanishing band gap. For example, the 100$\%$ Si-substituted graphene is a wide-gap semiconductor with a direct energy gap of 2.56 eV. Energy gaps are direct or indirect being determined by the highest valence and the lowest conduction state near the $\Gamma$ point. Their values decline with decreasing of the adatom concentrations and they are strongly depended on the adatom distribution configurations, e.g., $E_g^{d}=0.56$ eV for the meta-configuration (Fig. 6c) under the 50$\%$ substitution. Also, the zero-gap semiconducting behavior is revealed in the 50$\%$ ortho-case (Fig. 6b), where an anisotropic Dirac-cone structure appears at a certain $\mathbf{k}$-point between $\Gamma$ and K. Only one Dirac point intersects with the Fermi level so that its density of states vanishes there. This is responsible for the zero-gap semiconductor. When the substitution concentration declines, the unusual zero-gap semiconducting behavior appears, in which only one conduction Dirac point intersects with the Fermi level near the $\Gamma$ point, as shown in Fig. 6d–f. Second, the linear Dirac cone at $\Gamma$ point in Fig. 5a,b is seriously separated and distorted in Fig. 6b, or even thoroughly destroyed in Fig. 6a,c. Also, this Dirac cone structure is significant deformed without separation and slightly shifted to the valence band at $\Gamma$ point, as shown in Fig. 6d–f. Third, the valence and conduction bands near the Fermi level are initiated from the $\Gamma$ point, but almost independent of the M and K points. Finally, all the energy bands are mainly co-dominated by the Si-guest and carbon-host atoms under the high substitutions (Fig. 6a–c). However, these Si-guest and C-host atoms-co-dominated energy bands only exist at certain energies under the lower substitutions (Fig. 6d–f). Furthermore, the separated $\sigma$ bands at deep energies purely due to C-(2p$_x$, 2p$_y$) orbitals are absent. These results suggest the existence of quasi-$\sigma$ and quasi-$\pi$ bondings, respectively, originating from the (3s, 3p$_x$, 3p$_y$)-(2s, 2p$_x$, 2p$_y$) and 3p$_z$-2p$_z$ orbital hybridizations.

### Spatial charge density

The multi-orbital hybridizations in chemical bonds, which are responsible for the adatom-diversified geometric structures, electronic band structures and density of states, can be delicately identified from the spatial charge densities (${\rho }$’s) and their variations (${\Delta \rho }$’s) under the various modifications. The latter is obtained from the difference between the Si-adsorption/Si-substitution and pristine cases. A pristine graphene, as clearly shown in Fig. 7a, presents a very high carrier density between two carbon atoms (red region enclosed by a black rectangle), indicating a rather strong $\sigma$ bonding due to three C-(2s, 2p$_x$, 2p$_y$) orbitals on the honeycomb lattice. Such bonding is hardly affected by the Si adsorptions (Fig. 7b,d,f). Also, there exists the $\pi$ bonding near the plane boundary along the *z*-direction (area covered by a red rectangle). The 2p$_z$-2p$_z$ orbital hybridizations in C–C bonds might be drastically changed under the Si adsorptions. The charge distributions related to Si adatoms and C atoms along the *x*-, *y*-, and *z*-directions present obvious variations. The strong evidences are illustrated by ${\Delta \rho }$’s in Fig. 7c,e,g. The charge density is enhanced near the carbon atoms on the (*z*, *x*)- and (*z*, *y*)-planes (red regions). This result means that some electronic charges transferred from Si adatoms to C atoms. In addition to the *z*-direction, the important charge variations along the *x*- and $y-$directions survive between Si adatoms and carbon atoms/silicon ones, indicating the multi-orbital hybridizations in Si–C and Si–Si bonds. According to the direction- and position-dependent variations of charge densities, there exist (3s, 3p$_x$, 3p$_y$, 3p$_z$)-2p$_z$ and (3s, 3p$_x$, 3p$_y$, 3p$_z$)-(3s, 3p$_x$, 3p$_y$, 3p$_z$) complicated interactions in Si–C and Si–Si bonds, respectively.

The Si-substitution cases exhibit diversified charge densities compared with the pristine and Si-adsorption ones. For the 100$\%$ Si substitution, the Si–C bonds, which form a honeycomb lattice (Fig. 3a), present sufficiently high charge densities between two neighboring atoms (Fig. 8a). They are formed by the quasi-$\sigma$ bondings, in which the charge-density-dependent strengths are greatly reduced compared to the $\sigma$ ones in C–C bonds (Fig.  7a). This is consistent with the longer Si–C bonds and the shorter C–C bonds. Similar results are revealed in other substitution cases, e.g., ${50}$ $\%$ Si substitution under the ortho-, and meta-configurations (Fig. 8c,e), and ${20}$ $\%$ substitution in Fig. 8g. There also exist the quasi-$\pi$ bondings near the boundary. These present the non-well-behaved charge distributions compared with those of a pristine graphene (Fig. 7a). The obvious variations of charge densities on the (*z*, *y*)- and (*z*, *x*)-planes, being clearly show in Fig. 8b,d,f,h, suggest significant (3s, 3p$_x$, 3p$_y$)–(2s, 2p$_x$, 2p$_y$) and 3p$_z$-2p$_z$ orbital hybridizations in Si–C bonds. The coexistence of multi- and single-orbital interactions are further supported by the atom- and orbital-decomposed densities of states.

### Orbital-projected density of states

The main features of electronic band structures can be fully identified in the density of states. Special structures, the van Hove singularities, mainly originate from the critical points in the energy-wave-vector space, in which the band-edge states might belong to the local minima and maxima and the saddle points. In general, there are three kinds of novel structures, the V-shaped structure crossing at the Fermi level, logarithmically divergent peaks [$${E\sim \,}$$ $${-2.4}$$ eV and 1.8 eV] and shoulders [$${E\sim \,-3}$$ eV], as clearly observed in a pristine graphene (Fig. 9a). They are, respectively, due to the linear Dirac cone at the K/$$\Gamma $$ point in Fig. 5a,b, the saddle points at the M points, and the extreme points of parabolic dispersions at the $$\Gamma $$ point. Specifically, the former two structures are generated by the $$\pi $$ bonding of $$2p_z$$ orbitals (dashed red curve), whereas the initial $$\sigma $$ bands are closely related to the (2p$$_x $$, 2p$$_y $$) orbitals (dashed green and blue curves). The $$\pi $$-bonding structures are separated from those of the $$\sigma $$ bondings.

Apparently, a lot of van Hove singularities in the density of states are created by the Si adsorption in the $100\%$ double- and single-side cases, being obviously displayed in Fig. 9b,c, respectively. The finite density of states at the Fermi level is responsible for the conducting behavior, while the vanishing value in the pristine case (Fig. 9a) corresponds to a zero-gap semiconductor. The chemical bonding between Si and C atoms is responsible for the significant overlap of the valence and conduction bands and the creation of new energy bands (Fig. 5c,d). The significant contributions of carbon-2p$_z$ orbitals appear in the whole energy range of ${-6}$ eV ${\le \,E\le \,3}$ eV. Specifically, for that above ${-4}$ eV, the non-negligible contributions from the four Si-(3s, 3p$_x$, 3p$_y$, 3p$_z$) orbitals come into existence, respectively, indicated by the solid pink, green, orange and purple curves in Fig. 9b,c. The van Hove singularities from these orbitals are merged. This clearly indicates the p-sp$^3$ orbital hybridization in C–Si bonds. The multi-orbital hybridizations, which replace the 2p$_z$-orbital bondings on the graphene plane, are also confirmed by the previous charge density distributions (Fig. 7b–g). However, the $\sigma$ bonding of carbon atoms are hardly affected by the Si adsorptions, in which they are represented by the isolated shoulder structure below ${-4.2}$ eV (${-4.1}$ eV) in the Si-100$\%$ (Si-50$\%$) adsorption cases. When the concentration decreases, as shown in Fig.  9d, only a few of van Hove singularities at certain energies are created by Si-guest atoms. The seriously deformed valence V-shaped structure and two logarithmic divergent peaks at $-3$ eV and $-0.5$ eV come to exist, respectively, resulting from the significant distorted valence Dirac cone structure and two saddle points at the M point in Fig. 5e. The finite density of states at the Fermi level also exist; however, it is responsible for the metallic behavior. Furthermore, the $\sigma$ shoulder structure of C-(2p$_x$ and 2p$_y$) orbitals appears at $-3.5$ eV, indicating the stronger $\sigma$ C–C bonds (shorter C–C bond lengths) in the low concentration system compared with the $100\%$ adsorption cases.

The substitution and adsorption cases are thoroughly different from each other in the main feature of density of states. For the former chemical modifications, the number of electronic states, which is revealed in Fig. 10, vanishes within the specific band-gap region centered at the Fermi level. Most of the substitution configurations and concentrations correspond to the finite gap semiconductors, e.g., energy gaps due to the highest occupied valence state and the lowest unoccupied conduction one at the $\Gamma$ point under the 100$\%$ substitution (Fig. 6a) and meta-50$\%$ substitution (Fig. 6c). Only the ortho-50$\%$ case in Fig. 6b belongs to the zero-gap semiconductors with a seriously distorted Dirac-cone structure between the $\Gamma$ and K points. In the case of the lower concentration substitutions, the unusual zero-gap semiconducting behavior and the distorted valence Dirac cone structure (Fig. 6d), respectively, resulting in the almost negligible density of states at the Fermi level and the seriously deformed V-shape structure in Fig. 10d. The van Hove singularities can create many various special structures, namely, the obvious V-shape structure across ${E_F}$, strong shoulders/asymmetric peaks, and prominent symmetric peaks, as observed in the adsorption cases (Fig. 9). Apparently, the atom- and orbital-projected densities of states show that the contributions coming from the Si-3p$_z$ and C-2p$_z$ orbitals (solid purple and dashed red curves in Fig. 10a–d) appear simultaneously. Furthermore, the merged special structures are also revealed in the other orbitals, e.g., the interactions of Si-(3s, 3p$_x$, 3p$_y$) and C-(2s, 2p$_x$, 2p$_y$) orbitals. These results clearly illustrate the multi-orbital hybridizations of sp$^2$–sp$^2$ and p–p in Si–C bonds. That is to say, the Si–C bonds present quasi-$\sigma$ and quasi-$\pi$ chemical bondings. In addition, it should have the $\sigma$ and $\pi$ bondings in C–C bonds. The predicted orbital hybridizations are consistent with the spatial charge distributions in Fig. 8 and can account for the Si-substitution-enriched band structures in Fig. 6, respectively.

Scanning tunneling spectroscopy (STS) can provide sufficient information on the density of states at the Fermi level and the various van Hove singularities due to the valence and conduction bands simultaneously. High-resolution STS measurements are available for distinguishing the semiconducting and metallic behavior. Furthermore, they are very useful in identifying the close relations between the electronic energy spectra and the orbital hybridizations of the significant chemical bonds. Such experimental measurements have been successfully utilized to verify the band properties near the Fermi level and the dimension-diversified van Hove singularities in the graphene-related systems even in the presence of magnetic field, such as 2D few-layer graphene systems with the AB, ABC, AAB stackings^83,84^, 1D metallic and semiconducting carbon nanotubes^85,86^, and 3D Bernal graphite^87^. Apparently, the theoretical predictions on the Si-adsorption- and Si-substitution-diversified density of states in monolayer graphene systems, which could be examined by the STS experiments, cover the finite or vanishing density of states at the Fermi level, and the low- and middle-energy van Hove singularities. That is, such experimental examinations can provide the critical information on the multi-orbital hybridizations in the p–sp$^3$ or sp$^2$–sp$^2$ chemical bondings.

## Conclusion

The diverse structural and electronic properties of Si-doped graphene systems have been studied by the first-principles calculations. Apparently, the geometric structures, band structures, spatial charge densities and DOSs exhibit the rich and unique features, in which they are sensitive to the various adatom adsorption and substitution configurations. The critical multi-orbital hybridization mechanism and chemical bonding scheme are proposed to explain the Si-induced diverse physical and chemical phenomena. The calculated results clearly show that free carriers and energy gaps might be created by the Si-adsorptions and Si-substitutions, respectively. The Si-adsorbed graphene systems can remain in the planar graphene plane, in which the optimal position corresponds to the bridge site. The planar hexagonal structures clearly indicate a very small variation in the $\sigma$ bonds of C-(2s, 2p$_x$, 2p$_y$) orbitals; that is, these three orbitals do not take part in Si–C bonds. The Si–C bond lengths are ${\sim \,2.1}$–2.5 Å, possible to form the significant multi-orbital hybridizations in Si–C bonds. Via detailed analyses on the spatial charge densities, the spatial charge variations and the DOSs, the sp$^3$–p orbital hybridizations are deduced to dominate in the chemical Si–C bonds. Obviously, the three physical quantities are consistent with one another. As for the Si-substituted graphene systems, they can also remain in the planar geometric structures, as observed in the Si-adsorption cases. The Si–C bond lengths on the honeycomb lattice are about 1.62–1.83 Å, shorter than those in the Si-adsorbed graphene systems. This means that more C-orbitals are strongly hybridized with the four Si-orbitals. The theoretical predictions in the Si-diversified geometric structures can be fully verified by the STM experimental measurement.

The Si-adsorptions and Si-substitutions thoroughly alter the unique band structure of pristine graphene systems, especially for the zero-gap semiconducting behavior and linear Dirac cone made by the $\pi$ bonds of the C-2p$_z$ orbitals. In the $100\%$ Si double- and single-side adsorption configurations, the Si-adsorbed graphene systems are semi-metals with free conduction electrons and valence holes. The Dirac-cone structure near the $\Gamma$ point is seriously distorted after the Si adsorptions. There are more valence and conduction bands accompanied by various band-edge states in the whole electronic energy spectrum, e.g., the emergent low-lying energy bands along the K$\Gamma$ and M$\Gamma$ directions. However, the $\sigma$ bands, which arises from the C-(2p$_x$, 2p$_y$) orbitals, exhibit a rigid red shift of ${\sim \,1}$ eV and ${\sim \,0.5}$ eV for the $100\%$ adsorption configurations and the single-adatom adsorption configurations, respectively. The above-mentioned important results directly reflect the critical mechanisms, namely, the multi-orbital hybridizations of sp$^3$–p in Si–C bonds, sp$^3$–sp$^3$ in Si–Si bonds and sp$^2$–sp$^2$ in C–C bonds. Such chemical bonds consist of four Si-orbitals and one C-orbital, being closely related to the bridge-site adsorption positions and charge distributions of the separated orbitals. High-resolution ARPES measurements are very useful for verifying the low-energy valence bands crossing the Fermi level along K$\Gamma$ and M$\Gamma$ and the rigid $\sigma$ bands initiated from the $\Gamma$ point. On the other hand, most of Si-substitution cases result in the semiconducting behavior with the finite or vanishing band gaps. The Dirac-cone structure presents a deviation from the $\Gamma$ point, a strong distortion, or even a full destruction. The number of valence and conduction bands remains the same after the Si-substitutions. Besides, they are co-dominated by the Si-guest and C-host atoms. Apparently, the main features of the band structures in the Si-substituted graphene systems arise from the sp$^2$–sp$^2$ and p–p orbital hybridizations in Si–C bonds. The rich essential properties of the Si-adsorbed and Si-substituted graphene systems can be utilized for nanoscaled applications. The complete theoretical framework developed by the first-principle calculations can be particularly generalized for other emergent materials.

## Methods

The geometric structures and electronic properties of Si-adsorbed and Si-substituted graphene systems are thoroughly explored using the density functional theory (DFT) implemented in Vienna ab initio simulation package (VASP)^88,89^. The many-body exchange and correlation energies, which come from the electron–electron Coulomb interactions, are calculated from the Perdew–Burke–Ernzerhof (PBE) functional under the generalized gradient approximation^90^. Furthermore, the projector-augmented wave (PAW) pseudopotentials can characterize the intrinsic electron–ion interactions. As to the complete set of plane waves, the kinetic energy cutoff is set to be 500 eV, being suitable for evaluating Bloch wave functions and electronic energy spectra. A vacuum space of 10 Å is inserted between periodic images to avoid any significant interaction. The first Brillouin zone is sampled by $$ 9 \times 9 \times 1 $$ and $$ 100 \times 100 \times 1 $$ k-point meshes within the Monkhorst–Pack scheme for geometric optimizations and electronic structures, respectively. Such points are sufficient in obtaining the reliable orbital-projected DOSs and spatial charge distributions. The convergence for the ground-state energy is $$10^{ - 5} $$ eV between two consecutive steps, and the maximum Hellman-Feynman force acting on each atom is less than 0.01 eV/Å  during the ionic relaxations.

Via delicate VASP calculations on certain physical quantities, the critical physical and chemical pictures, i.e., the multi- or single-orbital hybridizations in chemical bonds due to C-host and Si-guest atoms, can be achieved under a concise scheme. They will be useful in fully comprehending the fundamental physical properties. These important concepts are obtained from the adsorption- and substitution-diversified geometric structures, carbon- and silicon-dominated valence and conduction bands, the total charge distributions and their drastic changes after adatom adsorption or guest-atom doping and the atom- and orbital-decomposed density of states through detailed analyses. Also, such physical quantities could shed light on the significant differences between the atom adsorptions and substitutions, such as the metallic or semiconducting behaviors, the normal and irregular electronic energy spectra, and the complicated van Hove singularities, being attributed to the diverse chemical bondings. The developed theoretical framework can be conceivably generalized to emergent 2D materials, e.g., the chemical adsorption and substitution in layered silicene^91^, germanene^92^ and tinene systems^93^.
